# COGNITIVE INTERFERENCE ACROSS THE LIFESPAN

**DOI:** 10.1093/geroni/igac059.2955

**Published:** 2022-12-20

**Authors:** Rachel Cole, Arturo Espinoza, Brooke Yeager, Nandakumar Narayanan

**Affiliations:** University of Iowa, Iowa City, Iowa, United States; University of Iowa, Iowa City, Iowa, United States; University of Iowa, Iowa City, Iowa, United States; University of Iowa, Iowa City, Iowa, United States

## Abstract

Mental processes that facilitate goal-directed behavior can be negatively affected by age-related biological and physiological changes. Such cognitive changes impact daily activities like driving and using the computer, which can in turn influence social relationships and capacity to work. Previous research has shown that older adults are more affected by interference than young adults. It is critical to understand how cognition changes across the adult lifespan, as cognition during middle age may be predictive of cognitive decline in older age. We evaluated performance on the MultiSource Interference Task (MSIT). The MSIT draws on Stroop, Flanker, and Simon-type tasks; it taxes interference resolution and typically results in interference-related slowing. We studied 60 individuals (32 adults 18–39 years old, 17 adults 40–59 years old, and 11 adults 60–99 years old). As expected, age had a significant effect on accuracy interference (difference in errors between interference and control trials). Surprisingly, however, young adults demonstrated the highest (worst) accuracy interference cost, while middle-aged and older adults had similar interference cost. Replicating previous findings, age group did not have an effect on reaction time interference cost (slowing of response between interference and control trials). It is notable that, in this study, older adults did not fare worse than middle-aged adults. These surprising findings challenge long-held theories that age negatively impacts interference resolution. Understanding differences (and similarities) between cognition in middle-age and older age will be critical for promoting healthy cognition throughout the lifespan, which benefits daily activities and quality of life.

